# Severe bronchiectasis and inflammatory lung disease in a patient with anorexia nervosa and severe and enduring malnutrition - a case report

**DOI:** 10.1186/s40337-020-00351-y

**Published:** 2020-11-18

**Authors:** Anna Saran, Firszt Oliver, Tomasz Łosień, Monika Kulig-Kulesza, Jolanta Myga-Porosiło, Ewa Kluczewska, Dariusz Ziora

**Affiliations:** 1 Department of Radiology and Radiodiagnostics in Zabrze, Medical University of Silesia, Katowice, Poland; 2 Department of Medical Rehabilitation, School of Health Sciences, Medical University of Silesia in Katowice, Katowice, Poland; 3 Department of Pulmonology in Zabrze, Medical University of Silesia, Katowice, Poland

**Keywords:** Anorexia nervosa, Emphysema, Bronchiectasis, Case report

## Abstract

**Background:**

Persistent structural changes of the lungs in anorexia nervosa (AN) patients are rarely described in contemporary medical literature. The objective of our paper is to report a rare case of severe bronchiectasis and inflammatory changes to the lungs resulting from chronic malnutrition in a AN patient.

**Case presentation:**

We describe a patient with severe inflammatory lung disease caused by malnutrition, resulting in persistent bronchiectasis accompanying AN. We performed an analysis of the patient’s medical records including radiological findings and laboratory results. A review of available literature shows very little data available on this topic.

**Conclusion:**

Bronchiectasis and other structural changes of the lungs are rare, but severe complications of severe, chronic malnutrition. As exemplified by our case report, they may require extensive differential diagnosis and pose a significant clinical challenge due to their non-reversible character. A successful treatment relies heavily on the patient’s compliance and may be hard to achieve. Clinicians managing patients with anorexia nervosa should be wary of early respiratory tract dysfunction-related symptoms and always consider malnutrition bronchiectasis as a differential diagnosis option.

## Introduction

The adverse effects of malnutrition resulting from AN (anorexia nervosa) affect every system of the human body. Abnormalities of the respiratory function in AN have also been researched in the past [1]. However, consequences of severe and enduring malnutrition are rarely described. Several mechanisms have been postulated to contribute to the development of bronchiectasis. These include i.e. air trapping and hyperinflation of the lungs [1, 2]. Coupled with an increased risk of infections due to the deterioration of the immune system [3–6], the authors point towards a multifactorial pathogenesis of structural lung changes in AN. From a clinical perspective, these can initially be often overlooked and not taken into consideration due to their rarity [7]. Furthermore, affected patients are likely to be at risk of further health complications including subsequent pulmonary infections. Our aim is to describe a rare case of severe bronchiectasis and inflammatory lung disease in a patient with AN resulting in recurrent infections of the respiratory tract, chronic dyspnea and prolonged hospitalizations.

## Case presentation

**Fig. 1:**
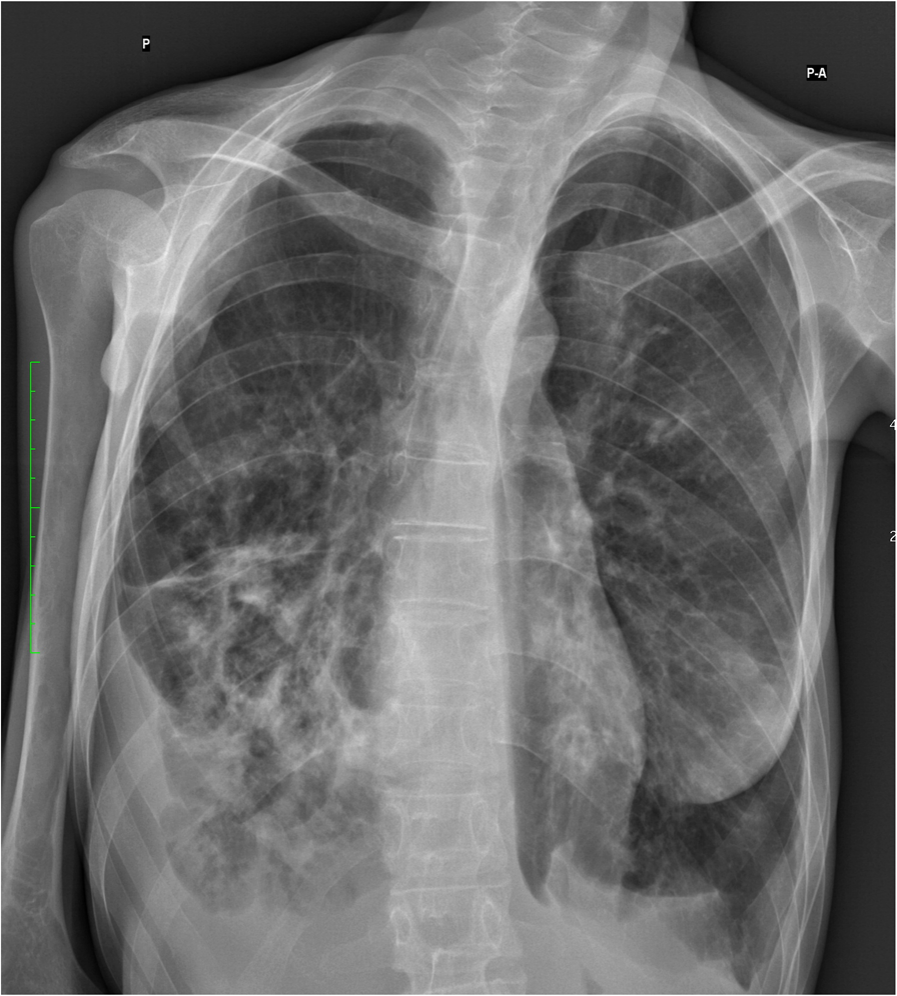
Chest X-ray on admission revealing multifocal consolidations mostly predominant in the right lung

In our paper, we describe the case of a 30-year-old woman with recurrent respiratory tract infections over the span of 3 years. She was first diagnosed with AN (restricting type) at the age of 16, and despite the physicians’ best efforts and the support of a clinical psychologist adequate cooperation and nutrition were extremely difficult to establish throughout her therapy. She had no history of smoking or drug abuse. In late 2017, the patient was referred to the Emergency Rescue Department (ER) by her general practitioner (GP) due to worsening symptoms of a respiratory tract infection. A week prior, initial symptoms included productive cough, fever and a mild dyspnea that were treated with antibiotics on an outpatient basis due to the patient’s refusal for hospitalization. On admission to the ER she complained of moderate dyspnea, chest pain, hemoptysis and productive cough. Her heart rate was elevated at 110 bpm and she had a decreased blood pressure value of 80/40 mmHg. Body temperature was normal, and she was fully alert and oriented. With a body weight of 23,5 kg and a height of 158 cm, her Body Mass Index (BMI) on admission was 9.2. Abnormalities in the lab results included an elevated C - reactive protein (CRP) level of 54.56 [mg/l, positive > 5 mg/l], decreased serum albumin (24.85 [mg/l, lab norm 35-40 mg/l) and a relatively low sugar level of 55.2[mg/dl]; pH level was 7.49. A chest X-ray revealed multifocal, ill-defined consolidations predominant in the right lung (Fig. 1).

**Fig. 2:**
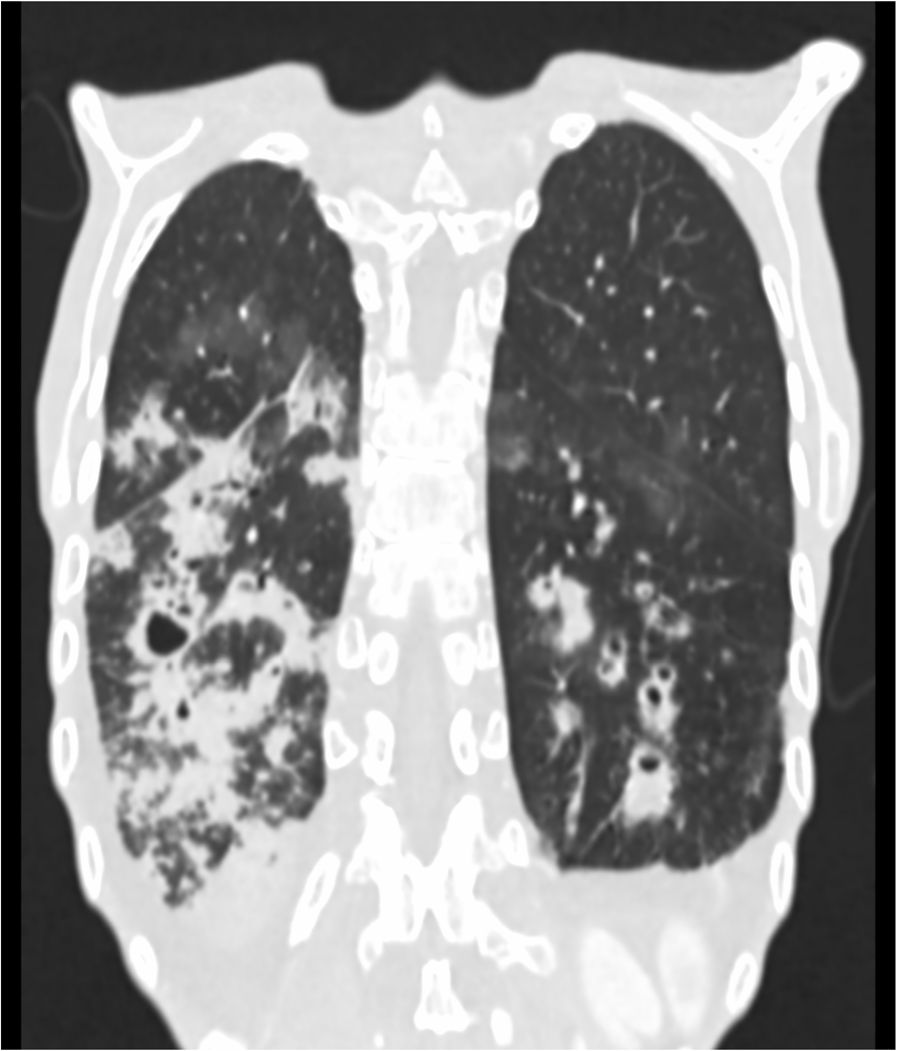
A CT scan of the chest revealed massive, cylindrical and saccular bronchiectases, with some segments of the bronchi filled with fluid content. Furthermore, diffuse areas of ground-glass opacification were also observed

She was admitted to the internal medicine ward and treated empirically with ceftriaxone and levofloxacin. Blood cultures and sputum samples were collected before antibiotic administration and came back negative a few days later. Bronchoalveolar lavage (BAL) samples collected during bronchoscopy came back negative, and tuberculosis was ruled out after several test panels; mycological cultures turned out negative as well. Cystic fibrosis was considered as another differential diagnosis option, but the tests had also negative results (CFTR gene test panel, twice). The patient’s condition stabilized after a few days of nutritional and antibiotic therapy, and she left the hospital at her own request shortly after refusing talks with a psychiatrist. She underwent further treatment on an outpatient basis. Due to the chronic nature of the patient’s ailment she was referred for a computed tomography (CT) scan of the chest. It revealed massive cylindrical and saccular bronchiectases, with some segments of the bronchi filled with fluid content. Furthermore, diffuse areas of ground-glass opacification were described in both lungs (Fig. 2). Unfortunately, the patient did not follow therapeutic recommendations or treatment of any kind.

**Fig. 3:**
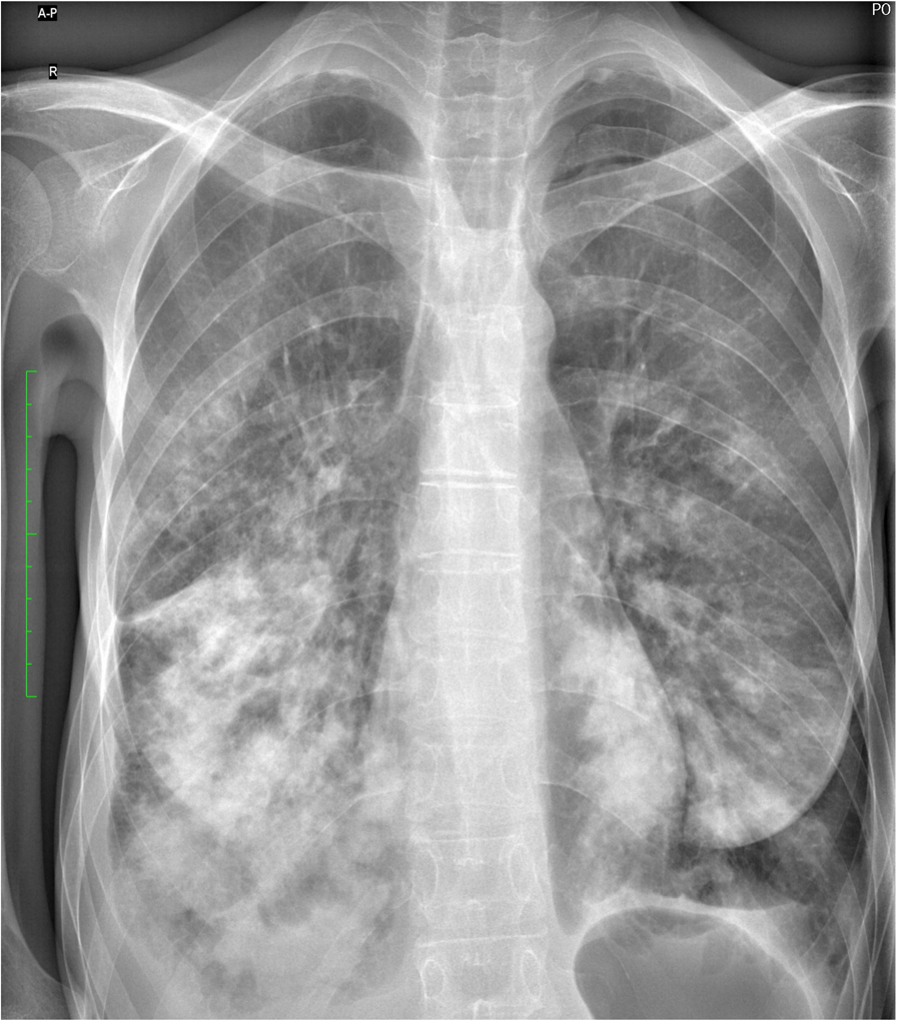
Subsequent chest X-ray revealing a similar state of the right lung compared to the previous examination

Sixteen months later, the patient was readmitted to the hospital due to another prolonged episode of pneumonia. She reported chronic cough in the past year and recurrent symptoms of respiratory tract infections, such as mild fever and periods of productive cough, exertion dyspnea and rhinitis. She was treated on an outpatient basis with clarythromycin without much effect. Chest X-ray on admission revealed once again diffuse consolidations, more dominant in the right lung (Fig. 3). Due to the worsening of the radiological image, another CT scan was performed to assess the lungs more precisely. Compared to the previous scan, a generalized progression of bronchiectases was observed with a notable enlargement of their diameter. Antibiotic therapy using ceftazidime and ciprofloxacin was used, and the extensive test panels repeated including tests against HIV, the flu virus, *Bordetella pertussis* and atypical pneumonia pathogens. Samples for these tests were collected before antibiotic therapy and all gave negative results. After 10 days of therapy and marked improvement of the patient’s general condition, she was discharged from the hospital and referred to a psychiatric ward for further treatment.

## Discussion

Persistent anatomical changes in the lungs have been described in the past in anorexia nervosa patients as a side effect of malnutrition [7]. However, literature on the topic is scarce and little data exists to fully explain the mechanism behind those changes. Some authors have also noted that the decreased strenghth of respiratory muscles in anorexia nervosa patients affects the residual volume of the lungs, which may promote the development of emphysema [8, 9]. Furthermore, severe weight loss has been associated with impairment of the diaphragm’ functioning, which may exacerbate dyspnea and respiratory failure during a respiratory tract infection [10]. It is also worth noting that the putative effect of low albumin levels, resulting in increased interstitial lung fluid and decreased lung clearance is another factor contributing to potential respiratory tract infections [11]. While several case reports and small studies have been published, their results are not conclusive and point towards a multifactorial pathogenesis of structural lung changes in AN [3–6].

Respiratory tract infections are a common problem and are one of the main reasons why people visit a general practitioner. Symptoms may include fever, cough, rhinitis, dyspnea, general fatigue and a wide range of changes in pulmonary auscultation. Many studies and case reports point towards the conclusion that AN patients are more susceptible to infections due to a weakened immune system [4, 12]. Furthermore, they are at a greater risk of rare complications, such as anatomical changes in the lungs [8].

In the described case, a state of chronic malnutrition was one of the key problems during treatment. Extensive efforts were made by her general practitioner (GP) and hospital physicians to convince the patient to pursue treatment, involving the patient’s family as well. While she was very open about her health issues with hospital staff and psychologists, she adamantly refused talks with psychiatrists. Her family remained supportive during each hospital stay, but all attempts to convince her to pursue further treatment have failed, with the patient claiming the problem to be of purely physical nature on the basis of her background in medical sciences. Our article highlights the need for alternate clinical management strategies, outside of traditional clinical approaches used in care of this patient. Unfortunately, evidence-based recommendations for preventing recurring malnutrition in AN patients are lacking, and further research is needed in this field as stated in a recent meta-analysis of articles concerning refeeding of malnourished patients [13].

## Conclusions

Persistent structural changes to the lungs are seldom, but severe complications of malnutrition in anorexia nervosa patients. Its multifactorial etiology makes it difficult to treat successfully, and it affects the patient’s quality of life significantly. Repeated diagnostic imaging should be considered in these patients in the event of recurrent and/or prolonged symptoms of respiratory tract infections. Chronic malnutrition affects the clinical outcome dramatically.

## Data Availability

No data and materials were generated during our study; we performed a retrospective analysis of the Patient’s medical records.

## Abbreviations

AN: Anorexia Nervosa
ER: Emergency Rescue Department
BMI: Body Mass Index
CT: Computed Tomography
GP: General Practitioner
BAL: Bronchoalveolar Lavage
